# A longitudinal investigation of the role of parental responses in predicting children's post‐traumatic distress

**DOI:** 10.1111/jcpp.12846

**Published:** 2017-12-02

**Authors:** Rachel M. Hiller, Richard Meiser‐Stedman, Sarah Lobo, Cathy Creswell, Pasco Fearon, Anke Ehlers, Lynne Murray, Sarah L. Halligan

**Affiliations:** ^1^ Department of Psychology University of Bath Bath UK; ^2^ Department of Clinical Psychology University of East Anglia Norwich UK; ^3^ School of Psychology and Clinical Language Sciences University of Reading Reading UK; ^4^ Department of Clinical, Educational and Health Psychology University College London London UK; ^5^ Department of Experimental Psychology University of Oxford Oxford UK; ^6^ Department of Psychiatry University of Cape Town Cape Town South Africa

**Keywords:** Longitudinal, child, post‐traumatic stress disorder, parenting, cognitive behavioural

## Abstract

**Background:**

While parental post‐trauma support is considered theoretically important for child adjustment, empirical evidence concerning the specific aspects of parental responding that influence child post‐traumatic distress, or the processes via which any such impacts occur, is extremely limited. We conducted a longitudinal examination of whether parental post‐trauma appraisals, trauma‐specific support style and general parenting style predicted child post‐traumatic stress symptom severity (PTSS) following trauma; and whether such influences operated via the child's own appraisals and coping style.

**Method:**

We recruited 132 parent–child pairs following children's experience of acute trauma. We examined whether parental responses assessed at 1‐month post‐trauma, predicted child PTSS at 6‐month follow‐up. Parental trauma‐specific appraisals and responses, and general parenting style, were assessed via both self‐report and direct observations. Child‐report questionnaires were used to assess PTSS and potential mediators.

**Results:**

Initial parent negative appraisals and encouragement of avoidant coping were associated with higher child‐reported PTSS at 6‐month follow‐up. Predictive effects were maintained even when controlling for initial child symptom levels. Observational assessments broadly supported conclusions from self‐report. There was evidence that parental influences may operate, in part, by influencing the child's own appraisals and coping responses. In contrast, there was no evidence for an influence of more “adaptive” support or general parenting style on child PTSS.

**Conclusions:**

Findings provide important insight into how elements of social support may influence child post‐trauma outcomes.

## Introduction

An estimated 10%–20% of children exposed to trauma will subsequently develop post‐traumatic stress disorder (PTSD), a potentially chronic problem with a number of negative consequences for children's developmental trajectories (Brewin, Dalgleish, & Joseph, 1996; Hiller et al., 2016). When considering children there is a critical need to take into account the developmental context in which trauma occurs, and particularly the role of the family environment in influencing children's adaptation following trauma (e.g. Cobham, McDermott, Haslam, & Sanders, 2016). In a meta‐analysis examining parenting behaviour as a predictor of child post‐traumatic stress symptom severity (PTSS) we found both negative (e.g. overprotection, hostility) and positive (e.g. support) aspects of general parenting behaviour to be reliably associated with children's PTSS, albeit accounting for a small proportion of variance (Williamson, Creswell et al., 2017). However, there was limited longitudinal evidence of parenting effects on child PTSS, which is problematic as the wider child anxiety literature highlights the possibility that parental behaviours (particularly overprotection) may be a response to child distress rather than a cause (e.g. Hudson, Doyle, & Gar, 2009). Moreover, the majority of studies utilised the same informant to measure both parenting and child PTSS, which may lead to overestimation of parental influences.

There are also key conceptual issues that require further examination. First, research has primarily focused on dimensions of parenting that are relatively broad (warmth, overprotection) and comparatively few studies have examined trauma‐specific parental responses (e.g. Cobham & McDermott, 2014; Gil‐Rivas & Kilmer 2013; Stallard, Velleman, & Baldwin, 2001). Theoretically, the latter are likely to be particularly important. Research suggests that parents identify and respond to trauma‐specific child needs (e.g. Alisic, Boeije, Jongmans, & Kleber, 2012; Prinstein, La Greca, Vernberg, & Silverman, 1996; Williamson, Creswell, Butler, Christie, & Halligan, 2016), and that aspects of parenting may change following child trauma (Cobham & McDermott, 2014). The limited available evidence suggests that trauma‐specific aspects of parental support, such as providing opportunities to talk about the trauma (Stallard et al., 2001), offering positive coping advice (Marsac, Donlon, Winston, & Kassam‐Adams, 2013) or reframing the trauma and its sequelae (Kilmer & Gil‐Rivas, 2010), could benefit child psychological outcomes. In principle, augmenting such aspects of parental responding could be one way of improving child outcomes following trauma, but further evidence is essential to inform this possibility.

A second issue is that there has been little consideration of the processes by which parental responses operate. Theoretical models of PTSD identify key cognitive behavioural processes that parents could potentially influence in children (Ehlers & Clark, 2000). Thus, negative appraisals of the self as vulnerable or permanently changed following the trauma are robust predictors of child PTSS (e.g. Meiser‐Stedman, Dalgleish, Glucksman, Yule, & Smith, 2009; Meiser‐Stedman, Smith et al., 2009), and the general anxiety literature highlights the potential for enhancement of child negative appraisals following parent–child conversations (Dadds, Barrett, Rapee, & Ryan, 1996; Percy, Creswell, Garner, O'Brien, & Murray, 2016). Although equivalent effects of parental appraisals in the context of child trauma have not yet been established, there is broader evidence that children's maladaptive appraisals may mediate associations between aspects of social support and PTSS (Hitchcock, Ellis, Williamson, & Nixon, 2015). Similarly, a tendency to engage in maladaptive coping styles (e.g. cognitive avoidance of trauma‐related material, rumination) may maintain or exacerbate child PTSS (e.g. Stallard & Smith, 2007). Again, the wider child anxiety literature indicates that parents of anxious children may encourage maladaptive ways of coping with feared stimuli, for example, by promoting child avoidance (e.g. Barrett, Rapee, Dadds, & Ryan, 1996; Dadds et al., 1996). However, whether such processes operate in a similar way in the context of child trauma remains to be demonstrated.

In sum, there is clear potential for parental responses to influence child PTSS. However, the available evidence precludes conclusions relating to which specific aspects of parental responding are important, or the psychological processes by which parents may influence children's adjustment. To address this, we conducted a longitudinal investigation of parental influences on child PTSS following acute trauma exposure. Specifically, we examined: (a) Trauma‐specific aspects of parental responses, namely the parent's own negative appraisals following the trauma (e.g. perceptions of child damage or vulnerability), and the extent to which they encourage negative or positive coping in the child (e.g. promoting avoidant coping vs. approach coping); (b) General parenting style characterised by overinvolvement/overprotection; and (c) The potential mediating role of children's own negative appraisals and maladaptive coping. Families were initially assessed 1‐month following child emergency department (ED) presentation, with follow‐ups 3 months and 6 months later. We hypothesised that 1‐month parental appraisals and parental support would be associated with child PTSS at 6‐month follow‐up, and that effects would be mediated, at least in part, via the child's own intervening maladaptive appraisals and coping behaviours, measured at 3 months. We expected that parental negative appraisals, promotion of avoidant child coping and general overinvolvement/overprotection would each predict higher levels of child PTSS, whereas parental support for approach coping in the child would predict lower subsequent symptoms. We assessed parenting domains via both self‐report and observational tasks, while child PTSS and mediating processes were measured via child report, wholly independent of parental responses. Crucially, we examined whether initial parental responses could predict later child symptoms even over and above trauma severity and initial child PTSS.

## Method

### Participants

Trauma‐exposed children and their parents were recruited across four hospital EDs in the United Kingdom. Dyads were eligible if they attended the ED following an acute trauma and children were 6–13 years old. Exclusion criteria were: developmental difficulties in the child that precluded mainstream schooling; injury resulting in a significant traumatic brain injury; suspicion of intentional injury by the child (self‐harm) or the parent. The final sample consisted of 132 dyads, representing 39% of the total children screened as eligible at recruitment sites. A full flow chart of recruitment is provided online (Figure S1).

### Procedure

Ethical approval was obtained from the University of Bath and Oxford A NHS Research Ethics Committees. Potential participants were identified by ED staff, and, given permission, the research team contacted families to confirm eligibility. Parents provided informed consent, while children provided informed assent. Home assessments were completed at 2–6 weeks post‐trauma (hereafter referred to as 1‐month) and 6 months later (i.e. 7 months post‐trauma), and a questionnaire only assessment was completed at 3‐month follow‐up. Key child self‐report questionnaire measures have been validated for use with children from 6 to 7 years old (Meiser‐Stedman, Smith et al., 2009; Steinberg et al., 2013). Nevertheless, to ensure clear understanding of the questions, particularly for younger participants, questionnaires were read out by the researchers when needed. All participants were invited to complete the 6‐month assessment, regardless of whether or not the 3‐month assessment was completed. Retention rates at 3 months were 84% (where questionnaires were generally completed via post), while at 6 months 96% of the original sample was retained. There was some evidence of selective attrition at 3 months, with noncompleters experiencing less severe events (triage rating: completers: *M *=* *1.87, *SD *= 1.02; noncompleters: *M *=* *2.44, *SD *= 1.19; *p *=* *.02). There were no significant differences between 3‐month completers or noncompleters for age or sex of child or symptom severity (*p*s* *> .22). We examined parental domains measured at 1 month as predictors of child PTSS at 1 month and 6 months, and utilised 3‐month assessments to assess intervening child maladaptive post‐trauma cognitions and behaviours.

### Measures

#### Descriptives

Demographic and trauma‐related information was obtained from ED notes and parent interview. Objective trauma severity for the child was assessed using triage category (nurse rating of urgency of care), which ranged from 1 (immediate care required) to 4 (less urgent).

#### Child report measures

##### Child PTSS

Children completed the child self‐report versions of the PTSD Reaction Index (PTSD‐RI; Steinberg, Brymer, Decker, & Pynoos, 2004). The PTSD‐RI assesses 17 DSM‐IV‐TR PTSD symptoms, with responses rated on a 5‐point Likert scale ranging from 0 = *none of the time* to 4 = *most of the time* (α* *= .89).

##### Child PTSD diagnosis

The Anxiety Disorder Interview Schedule‐PTSD Module (ADIS‐PTSD; Silverman, Albano, & Barlow, 1996) was administered to parents and children by trained researchers. The ADIS‐PTSD provides a well‐validated diagnostic tool for PTSD, based on DSM‐IV‐TR criteria. Diagnostic inter‐rater agreement was established on 25% of the interviews (*k* = 1.00). Thereafter, approximately every sixth diagnostic interview was discussed at a consensus meeting.

##### Child maladaptive appraisals

Children completed the self‐report Child Posttraumatic Cognitions Inventory (CPTCI; Meiser‐Stedman, Dalgleish et al., 2009; Meiser‐Stedman, Smith et al., 2009), rating their agreement with 25 items on a 4‐point Likert scale (1 = *don't agree at all* to 4 = *agree a lot;* α* *= .93). The CPTCI measures maladaptive appraisals relating to permanent and disturbing change (e.g. ‘My reactions since the event mean I have changed for the worse’) and being a fragile person in a scary world (e.g. ‘Anyone could hurt me’), and has established reliability and validity.

##### Child coping

Children were administered the Child Posttrauma Coping Questionnaire (CPCQ), a self‐report scale adapted from existing measures that indexes cognitive components of post‐trauma coping (Ehlers, Mayou, & Bryant, 2003; Stallard & Smith, 2007). The 11‐item measure includes items on rumination and cognitive avoidance, rated from 0 = *not at all or only one time* to 3 = *a lot of the time* (α* *= .89).

#### Parent Report Measures

##### Parental responses to the trauma

Parents completed the Parental Trauma Response Questionnaire (PTRQ), a self‐report measure of their appraisals relating to the child's trauma and their provision of trauma‐specific support to the child (Williamson, Hiller et al., 2017). The 30‐item appraisal scale comprises three subscales: (a) permanent change/damage to the child or family, (b) preoccupation with the child's vulnerability, and (c) self‐directed blame, rated on a scale ranging from 0 = *don't agree at all* to 3 = *completely agree*. The 20‐item support‐style scale comprises five subscales: (a) promotion of behavioural avoidance, (b) promotion of cognitive avoidance, (c) overprotection, (d) continuing normal routines, and (e) approach coping, rated on a scale ranging from 0 = *not at all* to 3 = *a lot*. Internal consistencies for all subscales ranged from α* *= .68 to .90, with only the pretrauma routines subscale having an *α* below .7.

##### Parental overprotection

Parents completed the Parental Overprotection Scale (POS; Edwards, Rapee, & Kennedy, 2010), a 19‐item self‐report measure of general parental behaviours that restrict children's exposure to situations perceived to be threatening. Items are rated on a 5‐point scale (0 = *not at all* to 4 = *very much*). The POS was initially validated for parents of preschool children, but has since been applied to parents of older children (Clarke, Cooper, & Creswell, 2013), and showed excellent internal consistency in our sample (α = .92).

#### Interaction Tasks^1^

##### Parent–child trauma narrative

Dyads participated in a joint trauma narrative task in which they were provided with a standard set of instructions that asked them to describe the trauma together in their own way, beginning just before the event happened and including anything they thought important. Instructions were kept general to promote a more naturalistic conversation regarding the event. There was no time limit. Following this, the researcher provided the parent with 13 prompt cards containing questions about the child's thoughts and feelings during and after the event in order to encourage discussion of internal states. Researchers left the room during the task, which was video recorded and transcribed verbatim.

##### Coding and reliability

A coding manual was developed based on well‐established work in the child anxiety field (Murray et al., 2014; Reese, Haden, & Fivush, 1993) and modified based on knowledge of risk factors for PTSD (e.g. promotion of avoidance). Transcripts were chunked, and individual parental utterances were coded. All coding was completed by a trained coder who was blind to other assessments. Initial blind double‐coding was performed on 25% of the transcripts to establish adequate inter‐rater reliability (intraclass correlations [ICC] are listed below with each code). Following this, every 10^th^ transcript was quality checked to ensure coding consistency. Codes were as follows:

1. Avoidance: statements in which the parent suggests/endorses cognitive or behavioural avoidance (e.g. child: ‘I don't ever want to go back to the tree’; parent: ‘Yes, no more climbing trees’; ICC = 0.93);
2. Approach: parent suggests or encourages a cognitive or behavioural approach response (e.g. child: ‘I don't want to drive on that road’; parent: ‘It's okay, we've driven on that road lots before, we'll be fine’; ICC = 0.93);
3. Negative appraisals: parent makes a negative appraisal of the trauma or sequelae, including: blaming the child; emphasising external threat or child vulnerability; or perceptions of permanent change (e.g. ‘You could have been killed!’, ‘I don't think we'll ever be the same’; ICC = 0.90);
4. Positive appraisals: parent's positive trauma appraisals, including: threat minimisation; lack of blame or criticism; autonomy appraisal; or endorsement of temporary, positive or no change (e.g. ‘You'll be back to your usual self when your leg is better’, ‘You coped really well’, ‘It was just an accident’; ICC = 0.90).

##### Anagram task

Dyads completed a mildly stressful anagram task, requiring the child to solve six pages of scrambled words in 7 minutes (Creswell, Apetroaia, Murray, & Cooper, 2013). In this task the anagrams are overly difficult relative to the child's age, and accompanying instructions describe the task as a test of children's problem‐solving abilities. Parents are given a stopwatch to monitor their time and the correct answers, and are told to assist their child only if they think he or she really needs help. The researcher left the room during this video‐recorded task.

##### Coding and reliability

Parental behaviours were coded for each minute of the interaction on a 1 = *behaviour not present* to 5 = *behaviour very much present* scale. A parental anxious involvement score was derived, based on average ratings for parental intrusiveness, expressed anxiety and encouragement of task avoidance. Again, all coding was completed by a trained rater who was blind to the other assessment outcomes, with 25% of the videos double coded to establish adequate inter‐rater reliability (ICC = 0.75–0.82). Every 10^th^ video thereafter was quality checked for coding consistency.

### Data analytic strategy

To address the primary question of whether parent responses in the acute post‐trauma period could predict child PTSS, we ran a series of separate linear regressions that examined both cross‐sectional associations between each of the parenting variables and initial child PTSS, and longitudinal associations between parenting and child PTSS at 6 months. In longitudinal analyses, we also tested whether parenting effects were present even when controlling for initial child PTSS severity. For these primary analyses, we explored the role of each parental response in a separate regression, to allow us to examine what specific appraisals or support styles may predict child outcomes. As PTSS scores were positively skewed, we applied a square‐root transformation. Next, where longitudinal associations between parental domains and child PTSS were identified (based on *p *<* *.05), we used the PROCESS Macro (Hayes, 2012) to examine whether children's own appraisals and coping responses at 3 months post‐trauma‐mediated effects, via bootstrapping analysis with 5,000 resamples. Due to more significant missing data for child 3‐month cognitive behavioural measures (CPTCI/CPBQ), where 1‐ or 6‐month scores were available we used these to impute 3‐month scores using expectation‐maximisation procedures. Age and sex of the child, along with trauma severity (triage category) were included as covariates throughout all analyses.

## Results

### Descriptive statistics

Sample characteristics are presented in Table 1. The sample comprised 132 children, aged 6–13 years, and their participating parent (predominantly mothers, aged 25–60 years). Index traumas were: motor vehicle accident (52%), other serious accidental injury (27%, for example, serious falls), acute medical episode (8%; for example, acute anaphylaxis), assault (2%) or other event (8%, for example, house fire, near drowning). The majority of children were given a hospital triage rating of 1, meaning they required immediate care. Twenty‐six per cent of children (*n *=* *34) met criteria for PTSD at 1‐month post‐trauma (in some cases without the requirement for symptom duration of at least 1 month, as insufficient time had elapsed post‐trauma at the time of assessment), and 10% (*n *=* *12) at 6 months. PTSS scores ranged from 0 to 51 (*M *=* *18.54, *SD *= 13.34) at 1‐month and 0–33 (*M *=* *12.86, *SD *= 11.88) at 6 months.

**Table 1:** Descriptive information

Demographic characteristics	Statistic (*N *=* *132)
Parent characteristics	
Age in years (*M [SD]*)	39.7 (7.0)
Proportion mothers	119 (90%)
Proportion married or cohabiting	97 (74%)
Education status: School until 16yo or younger	36 (27%)
Further education	50 (38%)
Higher education	46 (35%)
Child characteristics	
Age in years, *M (SD*)	9.87 (1.8)
Male	82 (62.1%)
Ethnicity – Caucasian	121 (91.7%)
Triage category	
1 (immediate attention required)	61 (46%)
2 (very urgent)	29 (22%)
3 (urgent)	26 (20%)
4 (less urgent)	16 (12%)
Days in hospital (Min–Max, *M* [*SD*])	0–28, 2.64 (4.83)
Days of school missed^a^ (Min–Max, *M* [*SD*])	0–28, 5.52 (5.98)
Proportion requiring ambulance/helicopter	90 (70%)
Proportion with head injury	33 (25%)

a Days of school missed represents days missed prior to their first assessment.

### Parenting and Child PTSS

A full correlation matrix showing associations between parenting variables is presented online (Table S2).

#### Parent trauma‐related appraisals

A series of regression analyses examined whether child PTSS could be predicted by parent post‐trauma appraisals, specifically: parental self‐report on the PTRQ of their perceptions of (a) permanent change, (b) child vulnerability and (c) self‐blame; and direct observations of (d) negative and (e) positive trauma‐related appraisals from the joint trauma narrative task. Results are presented in Table 2. Negative appraisals about the child being very damaged or vulnerable showed positive cross‐sectional associations with child 1‐month PTSS (controlling for age, sex, triage category). In addition, there were significant longitudinal associations from all 1‐month parental maladaptive appraisal domains (self‐report and observed) to 6‐month child PTSS, which were relatively large in magnitude (see Table 2).

**Table 2:** Results of regressions for parenting variables predicting initial child post‐traumatic stress symptom severity (PTSS), and PTSS at 6 months, controlling for age, sex and objective trauma severity in step 1

	1 month post‐trauma			6 months post‐trauma		
	*Std B*	*ΔF* (1,125)	*ΔR* ^2^	*Std B*	*ΔF* (1, 113)	*ΔR* ^2^
Parental appraisals						
PTRQ permanent change	.24**	7.19**	.05	.43***	25.7***	.18
PTRQ child vulnerability	.20*	5.34*	.04	.32***	13.5***	.10
PTRQ blame	.15†	2.88†	.02	.25***	7.56***	.06
Narrative negative appraisals	.07	0.60	.005	.21*	5.26*	.04
Narrative positive appraisals	−.04	0.19	.001	−.01	0.01	.00
Parental behaviours						
PTRQ behavioural avoidance	.23**	6.93**	.05	.27***	9.39**	.07
PTRQ cognitive avoidance	.17†	3.44†	.03	.19*	4.27*	.04
PTRQ overprotection	.17†	3.64†	.03	.18†	3.68†	.03
PTRQ approach	−.06	0.49	.004	−.09	0.93	.008
PTRQ maintain routines	.06	0.49	.004	−.03	0.08	.001
Narrative avoidance promotion	.08	0.80	.006	.09	0.85	.007
Narrative encouraging approach	−.05	0.38	.003	.004	0.002	.00
General parenting style						
Anagram anxious involvement	−.05	0.34	.003	−.06	0.43	.003
Parental overprotection scale	.11	1.64	.01	.14	2.17	.02

PTRQ is Parent Trauma Response Questionnaire. ^†^ *p* < .10; *p < .05; **p < .01; ***p < .001.

Importantly, when regressions were rerun additionally controlling for initial child PTSS, each of the maladaptive appraisal domains continued to predict 6‐month symptoms: parental perceptions of permanent change accounted for an additional 10% of variance (β* *= .32 *p *<* *.001), their preoccupation with the child's vulnerability explained 5% of variance (β* *= .23, *p *=* *.003), self‐blame explained 3% of variance (β* *= .18, *p *=* *.02) and total negative appraisals during the joint trauma narrative explained 4% of unique variance (β* *= .19, *p *=* *.02). In contrast, positive parental appraisals during the joint narrative task were not significantly associated with child PTSS either cross‐sectionally or longitudinally (see Table 2).

#### Parent support

We similarly examined parental support. First, we investigated potentially maladaptive aspects of support, specifically: parent self‐report on the PTRQ of (a) encouraging child behavioural avoidance, (b) encouraging cognitive avoidance, and (c) overprotection; and direct observations of (d) promotion of avoidant coping during the narrative task. As can be seen in Table 2, only parental reports of encouraging behavioural avoidance were positively, cross‐sectionally associated with child PTSS at 1 month. However, encouraging behavioural and cognitive avoidance each positively predicted 6‐month child PTSS. There were no significant effects for promotion of avoidance as assessed during the joint narrative task. When regression analyses were rerun controlling for initial child symptoms, a significant longitudinal association remained for parental promotion of behavioural avoidance, explaining 3% of unique variance (β* *= .17, *p *=* *.03). However, the predictive effect of cognitive avoidance promotion was no longer significant (β* *= .07, *p *=* *.41).

We also examined positive aspects of parental support: (a) maintaining normal routines and (b) approach coping on the PTRQ, and (c) facilitation of approach as measured by the joint narrative task. None of these positive parental responses were significantly associated with child PTSS (see Table 2).

#### General parenting style

Neither observed parental anxious overinvolvement (anagram task) nor self‐reported overprotection (POS) showed any association with children's PTSS (see Table 2 for full results).

#### The role of potential mediators: child‐reported appraisals and coping

We tested whether parental cognitive and behavioural responses to the child post‐trauma predicted later PTSS via intervening child processes: namely, children's own maladaptive trauma appraisals (CPTCI scores) and negative coping style (CPCQ scores) at 3 months. Given the null findings for positive aspects of parental support, mediation analyses focused on overall negative domains from the PTRQ – total combined scores on the negative appraisals scale and the total combined scores from the maladaptive support‐style scales. Preliminary correlational analyses showed that initial parental negative appraisals were associated with 3‐month child maladaptive appraisals, *r*_*p*_ = .34, *p *<* *.001, and negative coping style, *r*_*p*_ = .27, *p *=* *.001; and initial parental maladaptive support similarly predicted 3‐month child appraisals, *r*_*p*_ = .20, *p *=* *.03, and negative coping, *r*_*p*_ = .24, *p *=* *.008. In turn, 3‐month child maladaptive appraisals and negative coping style were each strong predictors of 6‐month PTSS: appraisals *r*_*p*_ **= .68, *p *<* *.001; coping *r*_*p*_ **= .59, *p *<* *.001. Separate mediation analyses were run for each potential pathway.

We first tested whether there was an indirect pathway from parental negative appraisals (1‐month post‐trauma) to 6‐month child PTSS via intervening child negative appraisals (3 months). A significant indirect pathway was identified (coeff = .16, 95% CI = 0.05, 0.29), although a significant direct pathway from parental appraisals to child PTSS remained (coeff = .12, 95% CI = 0.04, 0.18). When the same analysis was run with child negative coping as the mediator, there was similarly a significant indirect pathway (coeff = .11, 95% CI = 0.04, 0.19), but also a significant direct pathway from parental appraisals to child PTSS (coeff = .17, 95% CI = 0.06, 0.28). In a particularly stringent analysis, when we retested indirect pathways controlling for initial child PTSS, both were retained: indirect effect of parental appraisals via child appraisals (coeff = .10, 95% CI = 0.03, 0.20); via child coping (coeff = .05, 95% CI = 0.01, 0.10).

Next, we tested the indirect pathway from parental maladaptive behavioural response to child PTSS via child negative coping, which was significant (coeff = .25, 95% CI = 0.07, 0.44), with the direct effect of the parental variable on child PTSS no longer being significant in the same model (coeff = .23, 95% CI = −0.04, 0.51). Similarly, there was also a significant indirect pathway via child maladaptive cognitions (coeff = .23, 95% CI = 0.02, 0.47), with the direct path from parental behavioural response to child PTSS failing to meet significance in the same model (coeff = .24, 95% CI = −0.002, 0.49, *p *=* *.052). Finally, in this case, rerunning mediation analyses controlling for initial child PTSS eliminated both indirect pathways.

## Discussion

We examined whether parental responses may influence children's post‐trauma adjustment, based on both self‐report and observational tasks. Parental initial negative post‐trauma appraisals and encouragement of avoidant coping were robust longitudinal predictors of child PTSS at 6 months, even once initial child symptom levels were controlled for. There was also evidence that children's own cognitive behavioural responses to the trauma provide indirect pathways from parental responding to later child PTSS. We found no indication that more adaptive parenting behaviours or nontrauma‐specific parenting styles influenced children's psychological recovery.

The current findings in relation to parental post‐trauma appraisals were striking in their robustness. We found evidence for a detrimental impact of parents’ negative appraisals on child PTSS, based on both parental self‐report and on direct observation of parents discussing the trauma with their children. Appraisals relating to perceived child vulnerability or permanent damage, a focus on negative aspects of the event (e.g. how unsafe the child was, how significant the threat was), and a parental perception of (self) blame each significantly predicted child PTSS at 6 months. Importantly, this was the case even after controlling for initial child PTSS, suggesting that parental appraisals in the post‐trauma period are an active influence on children's longer term adjustment, rather than simply being a response to initial child distress. As symptoms are likely to be relatively stable by 6 months post‐trauma (Hiller et al., 2016), negative parental appraisals may be a marker for longer term child PTSS.

The wider developmental literature indicates that the way in which parents appraise an event and discuss it with their child can help children make meaning of the situation (Fivush, McDermott, & Bohanek, 2008). Children may be particularly reliant on their parents to make sense of events that are novel, frightening or complex, as is the case for many traumatic exposures. If parents express appraisals relating to children being permanently changed or vulnerable, or highlight particularly negative aspects of the trauma, this may influence children's own appraisals and exacerbate their posttraumatic distress. Consistent with this possibility, we found evidence that children's own maladaptive appraisals provide an indirect pathway from initial parent appraisals to later child PTSS. However, the association between parent appraisals and child PTSS was not wholly accounted for by child negative appraisals, therefore other intervening mechanisms are likely. Moreover, children's own maladaptive coping similarly provided a pathway from parental appraisals to child PTSS. In sum, results suggest several mechanisms by which parental post‐trauma appraisals may influence child adjustment, and these could include direct exacerbation of child distress, or other intervening mechanisms not studied here (e.g. unmeasured aspects of parenting or overall levels of family distress).

We also found that parental promotion of avoidant coping in the weeks following trauma predicted children's subsequent PTSS. Again, effects were not merely a consequence of parental reactions to initial levels of PTSS in children, as predictive effects were still present when initial levels of child PTSS were controlled for. This was specifically the case for behavioural avoidance – promoting avoidance of places, people or activities that could remind children of their experience. Avoidant coping is theorised as a key maintainer of PTSD as it prevents necessary processing of trauma memories and disconfirmation of maladaptive appraisals, and may directly increase distress (Ehlers & Clark, 2000). School‐aged children are likely to be dependent on their parents to determine the extent of their engagement with, versus avoidance of, trauma reminders. Our results add to the growing literature suggesting parents can actively influence how children cope following a traumatic experience (Cobham et al., 2016; Marsac et al., 2013; Williamson, Creswell et al., 2017; Williamson, Hiller et al., 2017), and suggest that parental involvement in relation to direct exposure to trauma cues may be particularly important. Consistent with such an interpretation, we found tentative support for the possibility that parent promotion of maladaptive coping influences later child PTSS via children's own adoption of maladaptive coping strategies. However, once again an alternative pathway, via children's appraisals, was also supported.

We found no evidence that parental encouragement of more adaptive coping, such as helping children engage with reminders of the event, trying to maintain pretrauma routines, or focussing on positive aspects of the event (e.g. good medical care), facilitates better child adjustment. Measuring positive behaviours posttrauma is complicated by the fact that parental failure to promote recovery in an active way may mean something very different for a child who is not at all affected by the trauma, versus a child who is manifesting clear distress. Nonetheless, we included these positive domains in our study as they are important when considering what guidance could be provided to parents of trauma‐exposed children. For example, if parents are to be advised not to support avoidant coping, it is essential to know that actively encouraging approach coping has no potential to do harm, as our results indicate.

We also examined general parenting style of parental overprotection/overinvolvement, which is assumed to limit child autonomy and promote anxiety (Hudson & Rapee, 2001). However, we found no evidence of associations with child PTSS. This is in contrast to the findings of our recent meta‐analysis (Williamson, Creswell et al., 2017), where such negative parenting styles showed a small but reliable association with child PTSS. The discrepancy may reflect methodological issues, where the use of cross‐sectional designs and a single informant may have inflated effects in some previous studies. Nonetheless, our observation that specific elements of parental posttrauma responses (vs. general aspects of parenting style) may be predictors of child PTSS is encouraging, as these may ultimately offer circumscribed intervention targets.

The current study has key strengths, including the longitudinal design, and the use of observational measures and both parent and child informants. Nevertheless, there are a number of considerations that may influence the generalisability of the findings. First, we relied on child self‐report of PTSS and mediating processes, which may have increased measurement error given the young age of our sample. At the same time, child self‐report is recommended for PTSS due to evidence that parents under‐report children's symptoms (Kassam‐Adams, Garcia‐Espana, Miller, & Winston, 2006; Meiser‐Stedman, Smith, Glucksman, Yule, & Dalgleish, 2007); and parents may not be aware of children's internal appraisals or cognitive coping styles. Crucially, the use of child self‐report to measure symptoms also allowed us to overcome single informant bias. Second, although our sample captured families across a range of socioeconomic backgrounds it was culturally and ethnically homogenous, and focussed on children who had experienced a single‐incident trauma, primarily accidental injury. Findings cannot necessarily be applied to samples where the child has experienced multiple traumas or nonaccidental trauma. Third, we had some evidence of selective dropout at the 3‐month assessment, and mediation analyses should be interpreted with this in mind. Fourth, it was outside the scope of this study to explore potentially relevant upstream parental factors, such as pretrauma parent–child relationship quality or mental health history, or parental trauma involvement and associated distress (Hiller et al., 2015). Such factors could influence parental cognitions and behavioural responses, and warrant investigation. Finally, of course, our sample is unlikely to capture families who are particularly unwilling to talk about their trauma.

To conclude, we found that negative parental appraisals of children's experience of trauma, and encouragement of avoidant coping, robustly predicted poorer child‐reported psychological outcomes 6 months following an acute trauma exposure. Associations could not be explained by trauma severity or initial child symptom severity. Results build on evidence highlighting the potential role parents can play in assisting their child to cope following trauma. Our findings evidence the particular importance of the parent's own interpretation of the trauma and of trauma‐specific elements of the support that they provide. Providing parents with information that addresses concerns about the potential psychological impact of the event on their child, and gives constructive advice about providing support following a single‐incident trauma, may be a relatively cost‐effective mechanism for improving children's post‐trauma outcomes as part of a stepped‐care approach.

**Key points**

- Parents are often the primary source of support for children following trauma exposure, yet it remains unclear how their response or behaviours may have an impact on the child's psychological adjustment.
- In a longitudinal study of 132 parent–child pairs, recruited after the child's attendance at hospital following an acute trauma, we found that parent responses predicted child post‐traumatic stress symptoms 6 months later.
- Parent appraisals around threat or the child being very vulnerable or damaged predicted child posttraumatic stress symptoms, from both self‐reported and observed parental responses. Self‐reported encouragement of avoidant coping also predicted poorer child outcomes.
- Parents can play a central role in influencing a child's post‐trauma coping and adjustment. Providing them with evidence‐based information on helpful (and potentially unhelpful) ways to support their child post‐trauma could be a useful ‘low‐dose’ option for improving children's outcomes.

## Supporting information


**Figure S1.** Flow chart of recruitment numbers.
**Table S2.** Correlation matrix for associations (r) between parental predictor variables.

## Note

1 Full task protocols and coding manuals are available via the corresponding author.
