# Supplementary material for: A longitudinal investigation of the role of parental responses in predicting children's post‐traumatic distress
Source: J Child Psychol Psychiatry. 2017 Dec 2;59(7):781–9. doi: 10.1111/jcpp.12846 (PMC6849512; doi:10.1111/jcpp.12846)
Supplement: Supplementary file 1 — Figure S1. Flow chart of recruitment numbers. Table S2. Correlation matrix for associations (r) between parental predictor variables. [file JCPP-59-781-s001.docx]

Additional Supplementary Information for: A longitudinal investigation of the role of parental responses in predicting children’s posttraumatic distress – by Rachel M. Hiller et al.

Supplementary Figure S1. Flow chart of recruitment numbers.

341 eligible families

6-month follow-up: *n* = 127

(96% of original sample)

- 2 families were unable to be contacted

- 3 families were no longer interested in

participating

3-month follow-up: *n* = 111

(84% of original sample)

Primary reason for non-completion of 3-month follow-up was that questionnaires were not completed within the timeframe (no later than 3-months post-T1 (±1mo)

Reasons for non-contact:

- 94 could not be contacted within 1-month

- 52 were not interested/too busy

- 2 believed it would be too distressing for

child

Drop-out primarily because appointment was cancelled and was unable to be rescheduled within required 1-month post-hospital timeframe.

Final Sample at T1: *N* = 132

Reasons for not participating

- 25 could not be contacted or have their

first assessment scheduled within 1-month

- 13 were not interested/too busy

- 5 believed it would be too distressing for

child

151 agreed to participate

194 agreed to be contacted by research team

Supplementary Table S2: Correlation matrix for associations (r) between parental predictor variables.

|  | 1. | 2. | 3. | 4. | 5. | 6. | 7. | 8. | 9. | 10. | 11. | 12. | 13. | 14. |
| --- | --- | --- | --- | --- | --- | --- | --- | --- | --- | --- | --- | --- | --- | --- |
| **Parental Trauma Response Questionnaire** | | |  |  |  |  |  |  |  |  |  |  |  |  |
| 1. Permanent Damage |  |  |  |  |  |  |  |  |  |  |  |  |  |  |
| 2. Preoccupation/Vulnerability | .68** |  |  |  |  |  |  |  |  |  |  |  |  |  |
| 3. Self-Blame | .57** | .56** |  |  |  |  |  |  |  |  |  |  |  |  |
| 4. Cognitive avoidance | .37** | .33** | .09 |  |  |  |  |  |  |  |  |  |  |  |
| 5. Behavioural avoidance | .69** | .59** | .41** | .53** |  |  |  |  |  |  |  |  |  |  |
| 6. Overprotection | .33** | .51** | .28** | .33** | .33** |  |  |  |  |  |  |  |  |  |
| 7. Approach coping | -.08 | .13 | .01 | -.07 | -.11 | .12 |  |  |  |  |  |  |  |  |
| 8. Continuing routines | -.05 | .05 | -.05 | .23* | .10 | .13 | .22* |  |  |  |  |  |  |  |
| **Joint narrative task** |  |  |  |  |  |  |  |  |  |  |  |  |  |  |
| 9. Positive appraisals | -.07 | -.10 | -.21* | -.002 | -.01 | -.18^+^ | .05 | .10 |  |  |  |  |  |  |
| 10. Negative appraisals | .25** | .21* | .09 | .25** | .27** | .06 | -.18* | -.05 | .54** |  |  |  |  |  |
| 11. Avoidance coping | .17^+^ | .26** | .20* | -.04 | .11 | .03 | <.001 | -.03 | -.03 | .007 |  |  |  |  |
| 12. Approach coping | -.06 | -.17^+^ | -.13 | -.02 | .03 | -.11 | -.05 | .002 | .25** | .09 | .07 |  |  |  |
| **Anagram Task** |  |  |  |  |  |  |  |  |  |  |  |  |  |  |
| 13. Anxious involvement | .03 | .05 | .01 | -.14 | .003 | .26** | -.16^+^ | -.10 | .19* | .21* | -.02 | .02 |  |  |
| **Parent Overprotection Scale** |  |  |  |  |  |  |  |  |  |  |  |  |  |  |
| 14. Overprotection | .55** | .57** | .43** | .40** | .59** | .64** | .02 | .10 | -.15 | .18^+^ | .09 | -.10 | .25** |  |
| *M* (*SD*) | 2.67  (4.67) | 13.13 (9.35) | 2.46  (3.63) | 0.96 (1.75) | 0.95 (2.02) | 8.35 (4.34) | 7.57 (2.88) | 8.72 (3.11) | 9.56 (8.61) | 11.44 (8.60) | 1.20 (1.73) | 1.16 (2.11) | 1.78 (0.37) | 29.33 (15.96) |

*Note.* ^+^ *p* < .10, * *p* < .05, ** *p* < .001
